# Review of the* Trifolium amabile* Complex in Peru, with the Description of a New Species

**DOI:** 10.1155/2016/5435781

**Published:** 2016-04-05

**Authors:** Eduardo Antonio Molinari-Novoa

**Affiliations:** “Augusto Weberbauer” Herbarium, Academic Department of Biology, Faculty of Sciences, La Molina National Agrarian University, Lima, Peru

## Abstract

Here, we describe* Trifolium absconditum *sp. nov., a new species of the* T. amabile* complex from South America. It differs from other Peruvian* Trifolia* of the complex by having smaller stipules, leaves, inflorescences, and floral pieces. A key for Peruvian species of the complex is presented, and typifications for them are made when necessary and material is available in Peruvian herbaria. Thus, the number of Peruvian species in the complex is elevated to three:* T. amabile*,* T. absconditum*, and a resurrected* T. peruvianum*. Finally, it is suggested that Chile must be excluded from the distribution of this complex.

## 1. Introduction

*Trifolium* L. is a temperate and subtropical genus comprised of about 238 herbaceous species [1]. Within it, an American complex of around 20 species, found on dry mountain slopes from Mexico to Argentina, is currently considered as a single entity:* Trifolium amabile* Kunth, understood in a wide sense [2, 3].

In Peru, the only recognized species of the* Trifolium amabile* complex is, indeed,* T. amabile*, a native caespitose herb; it is characterized with its nonrooting nodes, present in the introduced species of the genus occurring in the country,* T. repens* L. and* T. polymorphum* Poir. and for its conspicuously smaller leaflets than those of the also introduced* T. hybridum* L. [4, 5]. It also differs from* T. dubium* and* T. pratense*, other introduced members of the genus, because they have a bigger habit.

Previously, another species was recognised:* T. peruvianum*; however, some researchers [5, 6] put that name in synonymy with* T. amabile*, despite being a notably distinct plant and thus previously recognized [7, 8]. Species of this complex are taxonomically located in subgenus* Trifolium*, section* Involucrarium* Hook* ex* Lojac., and may be, as a group, the origin of all the section of American clovers [9, 10].

In the course of our investigations on Andean Peruvian rosids, we revised thoroughly the specimens deposited at Herbarium “Augusto Weberbauer” (MOL) in Lima, Peru, which referred to plants of the genus, and took special emphasis on the* T. amabile* complex species. Within it, the following species were recognized:* T. amabile*,* T. peruvianum*, and an unnamed species, characterized for its smaller habit evident in its narrower stipules, shorter leaflets, and smaller flowers. Despite the fact that* T. amabile* can be, in fact, highly variable, the consistency of the floral characters and their constant appearance at a definite range that did not overlap with those of the* T. amabile* sensu stricto nor with those of* T. peruvianum*, besides the occurrence at different altitudes of those species, convinced the author that a taxonomic difference could be established. A similar opinion is expressed in recent surveys on the genus [3] but without proposing nomenclatural acts [11]. Therefore, we are encouraged to publish ours, leaving non-Peruvian species out of the reach of this study. Here, we present the description of* Trifolium absconditum *sp. nov., along with descriptions of the other Peruvian species of this complex.

Additionally, a key for the Peruvian species of the complex is provided, all specimens consulted of the three involved taxa of the country are cited, and typifications of some taxa, when material is available in Peruvian herbaria, are made.

## 2. Materials and Methods

The specimens analysed are deposited in the cited herbaria. They were evaluated with a RadioShack digital-electronical caliper and studied under a Leica ZOOM 2000 stereoscope, and all the measures were repeated five times for each herbarium sheet. The characters evaluated are the ones used in the descriptions. Data were processed using Microsoft Excel. The photographs of specimens were taken with a Nikon D800E high-resolution camera. A survey to the Andean hills of Lima was made, in order to secure a complete specimen of* Trifolium peruvianum*.

The following Peruvian herbaria were consulted: “Augusto Weberbauer” (MOL), “San Marcos” (USM), and “Magdalena Pavlich” (HUPCH). Additionally, a photograph of a specimen from the Argentinian “Museo Botánico” (SI) was used, and the “Herbario Nacional” of Chile (SGO) was visited, in order to clarify the presence of this complex in the country.

Nomenclature follows the cited works [2, 3].

## 3. Results

We present a key to determine the Peruvian species of the complex. Also, full descriptions are provided.

*Key to the Species of the Trifolium amabile Complex in Peru*

(1) Stipules longer than 6,8 mm. Leaflets longer than 6,5 mm.
(2) Sepal lobes narrower than 0.7 mm, usually attaining that measure.
   
*Trifolium amabile* Kunth (I).
($2^{*}$) Sepal lobes around 1,05 mm wide.
   
*Trifolium peruvianum* Vogel (II).
($1^{*}$) Stipules shorter than or equal to 6,5 mm long. Leaflets up to 5,7 mm.
   
*Trifolium absconditum* Molinari (III).

*(I) Trifolium amabile Kunth [12, p. 503]*

≡ *Lupinaster amabilis* (Kunth) C. Presl [13, p. 47].
= *Trifolium hemsleyii* Lojac. [10, p. 143].* Trifolium amabile* var.* hemsleyii* (Lojac.) D. Heller & Zohary [14, p. 127].
= *Trifolium humboldtii* Spreng. [15, p. 213].
= *Trifolium mathewsii* A. Gray [16, p. 398].
= *Trifolium macrorrhizum* Ulbr. [17, p. 2].
= *Trifolium pauciflorum* Willd.* ex* Steud. [18, p. 707].
= *Trifolium reflexum* Cham. & Schltdl. [19, p. 576].* Trifolium schiedeanum* S. Watson [20, p. 339].

*Description*. Perennial herb with a caespitose habit. Root woody, pivoted, 2,5–3 cm of diameter. Stems are up to 80 cm long, usually attaining 40–60 cm. Stipule acute, of 7 or more mm long. Petiole scarcely pubescent. Leaflet pubescent along the central vein in the abaxial side, adaxially subglabrous, usually 1.5 × 0.9 cm^2^, with weakly serrate margins. Petiolule 0.8 mm long. Inflorescence racemose, umbellate or capitulescent, with 14–16 flowers. Flower bracteate, with a puberulent, 2,5 mm long pedicel and subtended by a filiform or slightly triangular bract of about the length of the flower. Calyx 5,5 mm long with lobes less than 0,6 mm of width in the basal part, costate, pubescent or glabrous, with subentire margins and attenuate apices. Corolla pink to red. Banner of 8 × 5 mm wide, with a shallow notch. Wing spatulate, 0,8 mm long. Keel spatulate, almost as long as the wings. The four latter petals are asymmetrically clawed. Androecium diadelphous; the sheath fused for a half its length, 4 mm long. Free stamen almost 3 mm long. Gynoecium sessile; subglabrous, sheltering each 1 to 4 ovules. Geographical distribution: Colombia, Ecuador, Peru, Bolivia, Chile, Argentina.

*Notes*. Its large stipules, narrow calyx lobes, and usually long peduncles, besides its occurrence in lower elevations (0–3800 m), put apart this species from the others.

*Select Specimens*. MEXICO. México. Toluca. In pratis. Without date.* Humboldt s.n.* (F, photo!) [isotype of* T. amabile*]. PERU. Áncash. Bolognesi. Near Chiquián. In sandy-rocky soil. Herb with pinkish flowers. Elev.: 3350 m. May 15th, 1950.* Ferreyra 7450* (MOL). Bolognesi. Near Chiquián. Flowers: pinkish. On clay-rock soil. Elev.: 3350 m. May 15th, 1950.* Cerrate 650* (USM). Huánuco. Rondos, near Huánuco. In fields. Pink flowered herb. Elev.: 2400 m. January 9th, 1954.* Woytkowski 715* (MOL, USM). Lima. Cajatambo. Without date.* Arredondo s/n* (MOL). Oyón. Oyón District. Oyón locality. Herb. 10°40′05′′LS, 76°45′44′′LW. Elev.: 3678 m. April 5th, 2003.* Huamán & al*.* 385* (HUPCH) Pasco. In Malanchaca (downroad from La Quinua to Salcachupán). In sandy-clay soil. Prostrate herb, with white flowers, slightly pinkish-purplish. Elev.: 3400 m. April 1st, 1948. Determined by A. Burkart.* Ochoa 340* (MOL).

**Figure 1:**
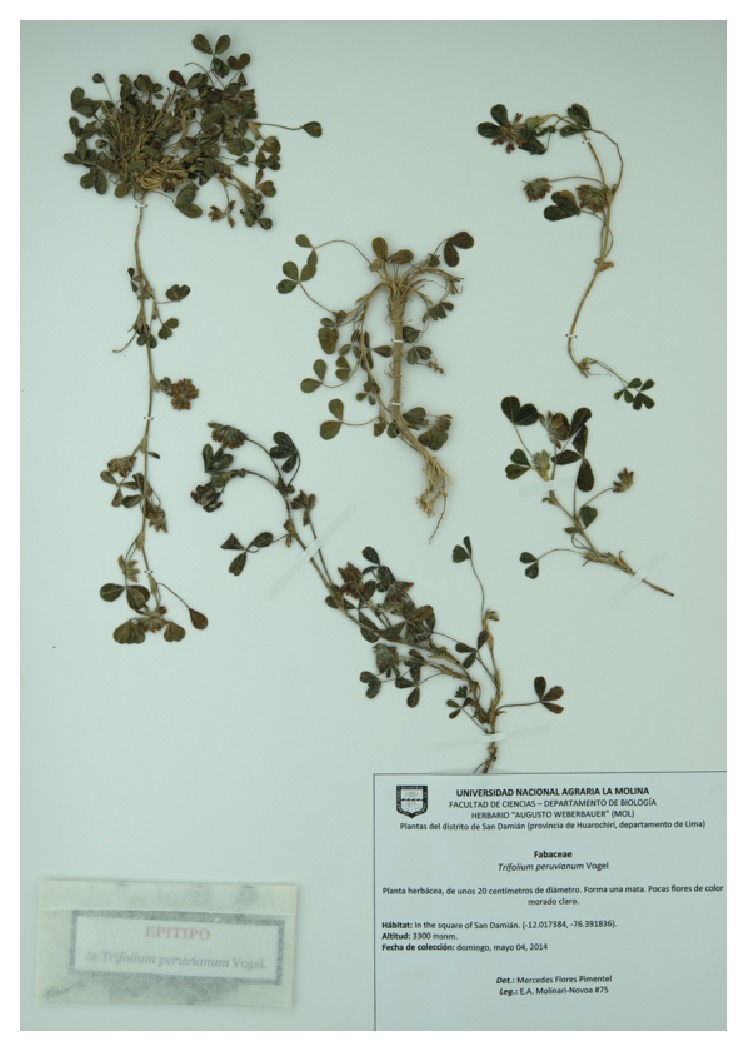
*Molinari-Novoa 75*: epitype of* Trifolium peruvianum* Vogel.

**Figure 2:**
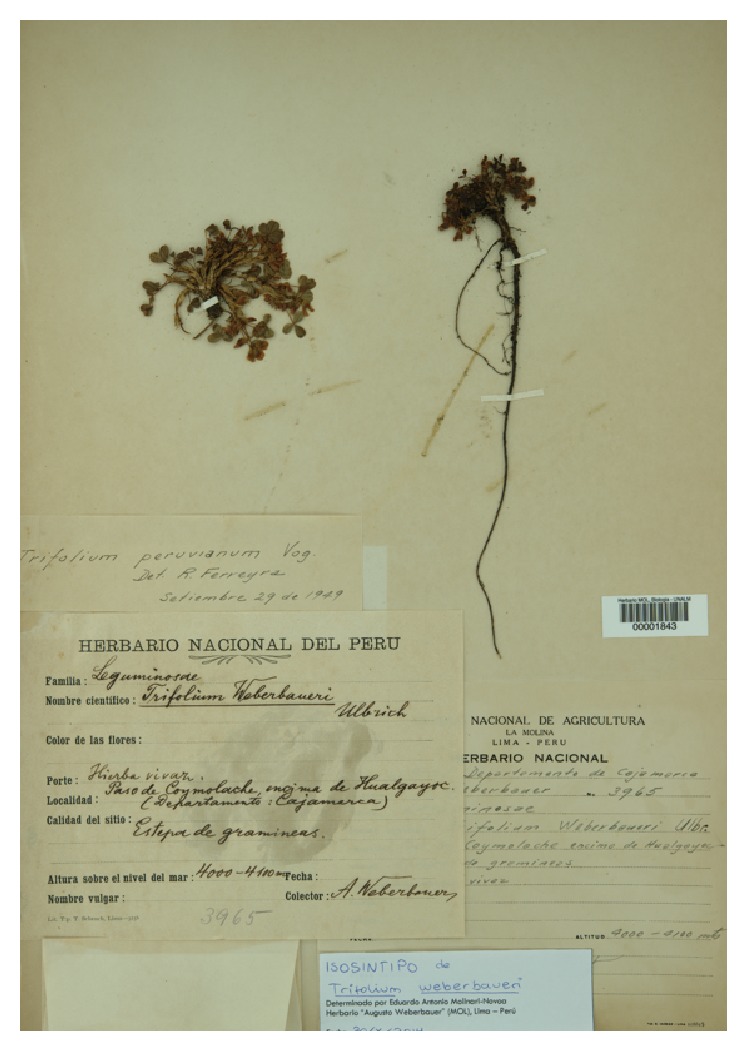
*Weberbauer 3965*: lectotype of* Trifolium weberbaueri* Ulbr. (=* T. peruvianum*).

*(II) Trifolium peruvianum Vogel (1843: 12) (Figures 1 and 2)*.* Lectotype* of* T. peruvianum* Vogel (*here designated*): PERU. Puno. Titicaca Lake. Elev.: 12 900 ft. July, 1831. Isosyntype of* T. peruvianum*.* Meyen s. n.* (F, photo!) Syntypes at B, destroyed.—*Epitype* of* T. peruvianum* Vogel (*here designated*): PERU. Lima. Huarochirí. In the main square of San Damián (−12.017384; −76.391834). Elev.: 3300 m. Sunday, May 4th, 2014.* Molinari-Novoa 75* (MOL!) (Figure 1).

= *Trifolium amabile* var.* pentlandii* Ball [21, p. 35].
= *Trifolium bolivianum* P. B. Kenn. [22, p. 97].
= *Trifolium chiclense* Ball [21, p. 35].* Trifolium peruvianum* var.* chiclense* (Ball) J. F. Macbr. [4, p. 451].
= *Trifolium weberbaueri* Ulbr. [17, p. 2].—*Lectotype* of* T. weberbaueri* (*here designated*): PERU. Cajamarca. Up from Hualgayoc. Near Coymolache Pass. Vivacious herb. In a grassland. Elev.: 4000–4100. May 11th, 1904. Isosyntype of* T. weberbaueri*. Weberbauer 3965 (MOL!). Syntypes at B, destroyed (Figure 2).

*Description*. Perennial, pubescent herbs, with a caespitose or cushion-like habit. Root woody, of almost 4,5 mm of diameter. Stems prostrate, usually growing near the root, up to 25 cm long. Stipules 0,7–1 cm long with acute apices. Petioles 1–6 cm long, pubescent. Leaflets elongate, obovate, 6–15 mm long, pubescent especially in the midrib abaxially, subsessile. Petiolule less than a millimetre long. Inflorescences axillary, racemose, subsessile due to the 1,5–4 mm long peduncle; often shorter than the subtending stipule, with 14–20 flowers per inflorescence. Flower subtended by a linear-filiform bract, with a pedicel up to 4 mm long. Calyx 5 mm long, with broad lobes of 0,7–1,5 mm wide. Corolla white, pinkish or purplish. Banner short and broad, 5 × 8 mm^2^. Wing with spathaceous lamina, 3,5–4 mm long, unguiculate, with a rounded apex. Keel slightly different than the wings, being 0,5 mm shorter or longer, also spatulate. Androecium diadelphous, the sheath 3,5–4 mm long, fused for a third of its length. Free stamen shorter, 2,5 mm long. Gynoecium 4,5 mm long, sessile with moderate pubescence, 1 mm long style, sheltering usually 2, up to 4, ovules. Geographical distribution: Ecuador, Peru, Bolivia, Argentina.

*Notes*. The usually long stipules, the wide calyx lobes, the long, wide petals, and the subsessile heads, besides its occurrence at higher elevations (2800–4500 m), set this species apart from* T. amabile*. An epitype is designated for* Trifolium peruvianum*, since the lectotype at F is fragmentary: a single leaf with a single flower, unfit for posing as a type [3].

*Select Specimens*. PERU. Áncash. Bolognesi. Mahuay. Pink flowers. In mountains' lower parts. Elev.: 4700 m. May 22nd, 1954.* Cerrate 2213* (USM). Cajamarca. Up from Hualgayoc. Near Coymolache Pass. Vivacious herb. In a grassland. Elev.: 4000–4100. May 11th, 1904.* Weberbauer 3965* (MOL) [lectotype of* T. weberbaueri*]. Huancavelica. Lircay. Rupacc. Whitish-pinkish flowers. (18L 0523300, 8556696). Elev.: 3957 m.* Castañeda 845* (MOL, USM). Junín. Conocancha. Natural grasslands. Elev.: 4100 m. February 11th, 1982.* Tiller & Maas 148*. Tarma. Grassland with sparese shrubs. Elev.: 3300–3700 m.* Weberbauer 2397* (MOL). La Oroya. Grasslands with sparse shrubs. Elev.: 3700–3800 m.* Weberbauer 2566* (MOL). Lima. Canta. Down road from Huavos town. Humid prairie, with other three species of* Trifolium*. Herbs with white flowers. Elev.: 3450 m. February 25th, 1992.* Flores & al. 7969 *(USM). Canta. Lachaqui, Taraca, on the highway to Arahuay. On clay-rock soil. Decumbent herb, with short peduncles. White-pinkish flowers. Common name: “trébol”. Elev.: 3500 m. April 12th, 2012.* Vilcapoma 7969* (USM). Huarochirí. In the Main Square of San Damián town (−12.017384; −76.391834). Elev.: 3300 m. Sunday, May 4th, 2014.* Molinari-Novoa 75* (MOL) [epitype of* T. peruvianum*]. Puno. “Pampa de Cuanhuilla”, a vast Ichu-tussock-grassland with few small round cacti,* Tetraglochin* (*strictum*?) and few other small shrubs, with calcareous outcrops above, in small valley on Puno-Arequipa road, at 50 km ca. 35 km (air) of Puno, 6 km SW of Manazo.* Solanum acaule*! Elev.: ca. 3800 m. January 11th, 1963.* Iltis & Ugent 1384* (MOL).

**Figure 3:**
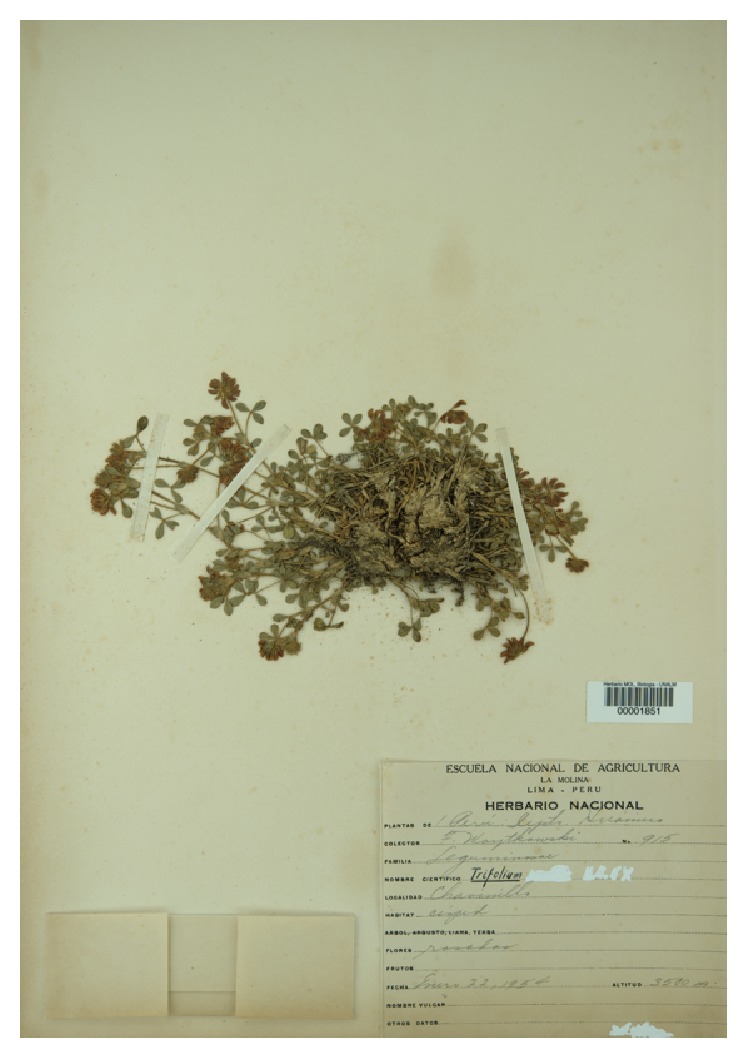
*Woytkowski 915*: holotype of* Trifolium absconditum* Molinari, sp. nov.

*(III) Trifolium absconditum Molinari*,* sp. nov. (Figure 3)*

*Holotype*. PERU. Huánuco. Chavinillo. In grasslands. Herb with pink flowers. Elev.: 3500 m. January 22nd, 1954.* Woytkowski 915* (MOL!)

 
*Dissimile T. amabili ac T. peruviano quoniam stipulæ minores*.

*Description*. Perennial herb, with a caespitose habit. Root pivoted, woody, of 5 mm in its wider point. Stem prostrate, 5–20 cm long, shorter in higher-altitudes plants. Stipule always present, 6,5 or less mm long. Petiole pubescent of 8–10 mm long. Leaflet obcordate, pubescent along the central vein in the abaxial side, adaxially subglabrous, usually 5 × 3 mm^2^, with the margins serrate. The central leaflet is slightly narrower and longer than the other two; Petiolule 0,5 mm long, subglabrous. Inflorescence racemose, capitulescent, axillar, subsphaerical, 1 × 1,2 cm^2^ of area, with 8–10 flowers each. Flower subsessile, with a subglabrous or sparsely pubescent pedicel of 1,5 mm long, and subtended by a triangular, very narrow bract. Calyx densely pubescent with hairs up to 0,5–0,75 mm long, all the structure 2,5–4,5 mm long, with the lobes triangular, each one with an acute apex and with entire margins. Corolla whitish, purplish or pink (exceptionally red). Banner obcordate, longer than wide, with an almost imperceptible notch, 5 × 4,5 mm^2^ or smaller. Wing about 4,5 mm long, spathaceous and asymmetrically clawed with a rounded apex each. Keel slightly shorter, than the wings, with rounded-acute apices. Androecium diadelphous, with the connate stamens fused for a third of their length attaining 4 mm long. Free stamen shorter, up to 2,5 mm long. Gynoecium with a 2-3 mm long ovary, puberulent; style subglabrous, 1 mm long, stout. The gynoecium shelters 2 ovules.

*Etymology*. This species is so called since it has been commonly deposited in herbaria, but without being described all along. Geographical distribution is as follows: Peru and Argentina. 

*Notes*. It can be easily recognized by its smaller overall aspect, with less and sparser flowers per inflorescence and the shorter stipules in mature leaves. 

*Paratypes*. PERU: Áncash. Bolognesi. Pagchu, Chilcas valley. Pink flowers. Low mountain. Elev.: 3600 m. May 2nd, 1978.* Cerrate 7198* (USM). Cuzco. Yaurisque. Paruro. SW from Cuzco. From Cuzco to Paruro. Prostrated herb, red-white flowers.* Núñez 7394* (USM). Huánuco. Huánuco: Chavinillo. In “puna” formation. Herb with purplish flowers. Elev.: 3800 m. January 12th, 1954.* Woytkowski 1040* (MOL). Junín: La Oroya. Grassland with sparse shrub. Herb Elev.: 3700–3800 m.* Weberbauer 2566-A* (MOL). Moquegua. General Sánchez Cerro. Ubinas District. Colpamayo site, Tassa Locality. Small plateau with shrub vegetation. Herb, creeping, trifoliate 1.* Montesinos 2007* (HUPCH). ARGENTINA. Salta. Chicoana: Ruta 33, from Cachi to Ciudad de Salta, after detour for Ruta 42. February 15th, 2002.* Cialdella & al. 310* (SI, digital image!).

## 4. Discussion

*Trifolium weberbaueri* is rather problematic, since the description of the types refers to small plants (as the lectotype itself), which can fit in either* T. absconditum* or* T. amabile* if evaluated using vegetative characters [17]. However, the flower proportions (especially those of the wide banner petals) and the stipules' length of the isosyntype here lectotypified clearly set the species under synonymy with* T. peruvianum*, as recently suggested [2, 3].

“*Trifolium amabile* var.* pedicellaris* Ball” is a commonly seen trinomial in Peruvian herbarium sheets; however, no bibliographic references were found for it. Anyway, the plants identified as such are consistently within our definition of* T. peruvianum*.

All species inhabit sandy soils; however, some specimens are cited for rocky or clay soils. The author had observed that species of this complex are also opportunistic, thriving in gardens and fodder pastures. In Peru, these plants are called “chullasapi, chullachaqui” (meaning “with one foot,” referring to the long, deep, pivoted root; the former name is a corruption of the latter). It is used as a medication against lungs illness. The root is grinded, boiled, and then consumed; or it can be chewed raw (R. Castañeda, pers. comm.)

Chile was included as a country where* T. amabile* do occur [14]. This seemed quite probable, although no other bibliographic reference or a specimen from there has been located. However, after a visit to the SGO, it became clear that no specimens of the complex are present; and thus, Chile should be excluded from the range of the complex until further notice.

Albeit the fact that discrete characters are recognized and we are sure morphometric approaches to the* Trifolium amabile* complex have been successful and appropriate, we strongly suggest molecular examinations of the validity of all taxa complex, including both Northern and Southern American species. Despite the works recently done by the cited American authors and the present work, increasing the quantity of recognized species of American trefoils while reducing the number of valid names, only genetic research will put beyond doubt all the valid species and therefore clarify definitively the taxonomy of this group.
